# Clinical implications of preoperative echocardiographic findings on cardiovascular outcomes following vascular surgery: An observational trial

**DOI:** 10.1371/journal.pone.0280531

**Published:** 2023-01-19

**Authors:** Matthew J. Meyer, Slater A. Jameson, Edward J. Gillig, Ankur Aggarwal, Sarah J. Ratcliffe, Mary Baldwin, Karen E. Singh, W. Darrin Clouse, Randal S. Blank

**Affiliations:** 1 Department of Anesthesiology, University of Virginia School of Medicine, Charlottesville, VA, United States of America; 2 Department of Anesthesiology, Newton Wellesley Hospital, Newton, MA, United States of America; 3 Department of Surgery, Franciscan Physicians Network Vascular Surgeons, Indianapolis, IN, United States of America; 4 Department of Public Health Sciences, University of Virginia School of Medicine, Charlottesville, VA, United States of America; 5 Department of Surgery, University of Virginia School of Medicine, Charlottesville, VA, United States of America; Cleveland Clinic, UNITED STATES

## Abstract

**Introduction:**

Peripheral artery disease and cardiac disease are often comorbid conditions. Echocardiography is a diagnostic tool that can be performed preoperatively to risk stratify patients by a functional cardiac test. We hypothesized that ventricular dysfunction and valvular lesions were associated with an increased incidence of expanded major adverse cardiac events (Expanded MACE).

**Methods and materials:**

Retrospective cohort study from 2011 to 2020 including all patients from a major academic center who had vascular surgery and an echocardiographic study within two years of the index procedure.

**Results:**

813 patients were included in the study; a majority had a history of smoking (86%), an ASA score of 3 (65%), and were male (68%). Carotid endarterectomy was the most common surgery (24%) and the least common surgery was open abdominal aortic aneurysm repair (5%). We found no significant association between the echocardiographic findings of left ventricular dysfunction, right ventricular dysfunction, or valvular lesions and the postoperative development of Expanded MACE.

**Conclusions:**

The preoperative echocardiographic findings of left ventricular dysfunction, right ventricular dysfunction and moderate to severe valvular lesions were not predictive of an increased incidence of postoperative Expanded MACE. We identified a significant association between RV dysfunction and post-operative dialysis that should be interpreted carefully due to the small number of outcomes. The transition from open to endovascular surgery and advances in perioperative management may have led to improved cardiovascular outcomes.

**Trial registration:**

**Trial Registration:** NCT04836702 (clinicaltrials.gov). https://www.google.com/search?client=firefox-b-d&q=NCT04836702.

## Introduction

Peripheral artery disease and cardiac disease are often comorbid conditions with similar risk factors [1]. In the vascular surgery population, the prevalence of coronary artery disease, congestive heart failure, and valvular disease is 50% [2,3], 15% [3], and 7% [3], respectively. With the incidence of post-operative myocardial ischemia and myocardial infarction approaching 46% and 5% respectively [4], the importance of perioperative cardiac risk stratification and associated surgical optimization is vital.

Validated models to predict postoperative morbidity and mortality exist. The American College of Surgeons’ National Surgical Quality Improvement Program Surgical Risk Calculator [5] is designed for a myriad of surgeries and outcomes. The Revised Cardiac Risk Index (RCRI) [6] is designed to be easily applied to assess cardiac risk in any patient. The heterogeneity of these models renders them less accurate in predicting cardiac outcomes in vascular surgery patients [7] than vascular specific models like the Vascular Study Group of New England Cardiac Risk Index [8]. Still such calculators are not without limitations [9], relying heavily on medical history, rather than dynamic clinical assessments of cardiac function.

Transthoracic echocardiography is non-invasive, inexpensive, and can be quickly performed by experienced providers at bedside. It has ideal qualities for perioperative risk assessment, even in an urgent setting. Moreover, studies have linked abnormal echocardiographic findings with increased risk of adverse cardiac events for non-cardiac surgeries [10–12].

While current American Heart Association /American College of Cardiology perioperative guidelines acknowledge the utility of echocardiography in limited cases when there is a concern for pulmonary artery hypertension or moderate to severe valvular pathology [13], echocardiography has not been officially incorporated into any widely used perioperative risk algorithm. Even the new vascular quality initiative cardiac risk index does not take into consideration potentially valuable and often readily available echocardiographic results [14].

We designed our study to address the clinically important question of which echocardiographic parameters, if any, should be considered predictively useful for patients undergoing vascular surgery. This single center, retrospective cohort analysis investigates the potential of echocardiographic parameters obtainable by the point-of-care ultrasound user to predict adverse cardiac outcomes for patients undergoing vascular surgery. We hypothesized that: 1) left ventricular dysfunction is associated with an increased incidence of expanded major adverse cardiac events (Expanded MACE), 2) right ventricular dysfunction is associated with an increased incidence of Expanded MACE, and 3) moderate or severe valvular lesions are associated with an increased incidence of Expanded MACE.

## Materials and methods

We designed and performed a retrospective, observational cohort study including all patients from a major academic center who had vascular surgery and an echocardiographic study within two years of the index surgery. A statistical analysis plan was written, and this study was registered on clinicaltrials.gov (NCT04836702; April 8, 2021; Matthew Meyer) prior to any data analysis. The dataset analyzed in this study is available from the corresponding author on reasonable request.

### Study approval and study population

This study was approved, and written informed consent waived, by the University of Virginia Health Science Research Institutional Review Board (#20906). Inclusion criteria were patients over the age of 18 undergoing vascular surgical procedures at the University of Virginia Medical Center between January 1, 2011, and June 23, 2020. The vascular surgical procedures included were: carotid endarterectomy (CEA), open abdominal aortic aneurysm repair (Open AAA; excluding any abdominal aortic surgeries with a thoracic aorta component), endovascular abdominal aortic aneurysm repair (EVAR), complex endovascular repair of aneurysm/thoracic endovascular aortic repair (Complex EVAR/TEVAR), suprainguinal arterial reconstruction (Supra), and infrainguinal arterial reconstruction (Infra). Arteriovenous fistula creation, extremity amputation, and open thoracic aortic surgery were excluded. Vascular surgeries were also excluded if their indication was infection, pseudoaneurysm or revision. Patients who underwent multiple included surgeries within 30 days were counted as a single data point and were classified with regards to the initial procedure. If an individual patient had multiple vascular surgeries more than 30 days apart, each procedure was considered as a separate index procedure.

Demographic data, medical history, intraoperative data, and in-hospital outcomes were extracted from UVA Health’s Vascular Quality Initiative database. Intraoperative data (eg. vasopressors and fluid volumes) were extracted from the UVA Health Perioperative Informatics database. Echocardiographic findings were manually extracted from the UVA Health electronic medical record (Epic) and stored in a novel database (Redcap).

### Surgical case complexity

We obtained data classifying surgical complexity. These included American Society of Anesthesiologists’ Physical status classification grade, amount and type of intraoperative fluids administered, estimated blood loss (EBL), and intraoperative vasopressor infusion requirement.

### Echocardiogram data

Echocardiogram data was obtained via electronic chart review at UVA Health (Epic EMR; Verona, WI, USA). The records of each patient were searched for transthoracic echocardiograms or transesophageal echocardiograms results from studies conducted within a two-year period prior to the patient’s index procedure. In cases with multiple studies conducted within that time frame, data from the most recent study was collected. If information was missing from the most recent echocardiography report (e.g., focused exam assessing for presence of thrombus), additional data was obtained and included from the next most recent study. Data obtained included: qualitative and quantitative classification of left and right ventricular ejection fraction, presence, and severity of left and right ventricular hypertrophy and cavity dilation, presence and severity of valvular dysfunction (aortic stenosis/regurgitation, mitral stenosis/regurgitation, and tricuspid regurgitation), and global qualitative assessment diastolic dysfunction.

### Outcomes

The primary outcome of this study was the compound outcome of Expanded MACE [15] within the postoperative admission. Our definition of Expanded MACE included the variables of non-fatal cerebrovascular accident, non-fatal myocardial infarction, new onset arrhythmia, congestive heart failure, and cardiovascular death. Secondary outcomes include the individual components of Expanded MACE along with new dialysis, respiratory complications, in-hospital mortality, and discharge disposition.

### Exposure groups

LV dysfunction was classified if an echocardiography report included either: 1) LV ejection fraction was calculated as <50%, 2) mild, moderate or severe decrease in LV systolic function, or 3) mild (grade 1), moderate (grade 2), or severe (grade 3) decrease in LV diastolic function.

RV dysfunction was classified if an echocardiography report included mild, moderate or severe decrease in RV systolic function.

**Fig 1:**
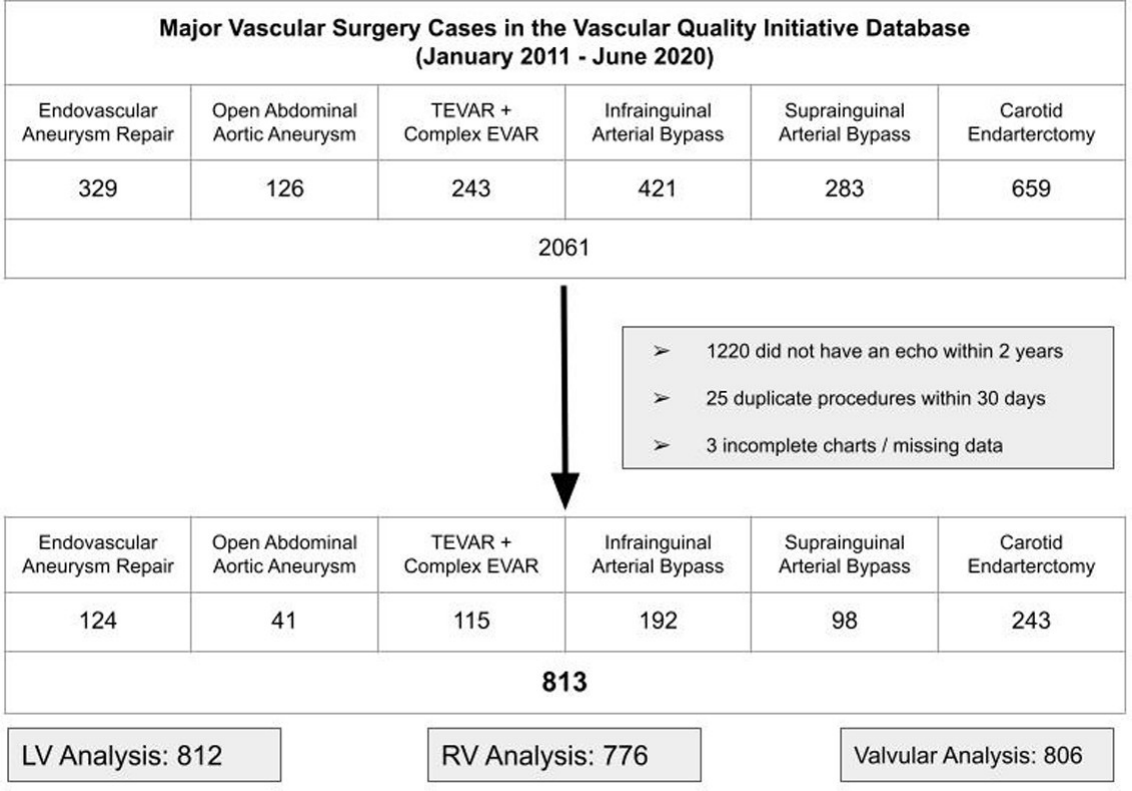
Inclusion and exclusion for study sample. EVAR: Endovascular aneurysm repair. LV: Left ventricular. RV: Right ventricular. TEVAR: Thoracic endovascular aortic repair.

Valvular pathology was classified if an echocardiography report included mitral stenosis, mitral regurgitation, aortic stenosis, aortic regurgitation, or tricuspid regurgitation that were qualified as either moderate or severe (Fig 1).

### Statistical methods

The statistical analysis plan was written *a priori* and published on clinicaltrials.gov (NCT04836702). Standard descriptive statistics were used to describe baseline characteristics at the time of surgery, both overall and within each exposure group. Summary statistics such as medians, ranges, minima and maxima, and percentages were produced for measured variables. Frequencies were computed for categorical and ordinal variables.

The primary outcome was first compared within each exposure group using logistic regression. Multivariable logistic regression was planned to test for associations after adjusting for other variables. Penalized logistic regression (lasso) was planned to identify variables for inclusion in the adjusted models. However, due to the small number of events observed, only unadjusted logistic or Poisson (for rare events) regression could be performed.

Before any regression models were constructed, all variables were checked for collinearity with each other and the exposure variables using the condition index and Spearman correlation matrix. Any variable deemed to be strongly correlated (rho>0.7) with an exposure variable was considered for removal from the potential set of adjustment variables. All other variables were allowed to enter the models.

Since there are three primary hypotheses of interest, an alpha level of 0.01 was used for statistical significance for the associations between the exposures and the outcome. A prospective sample size calculation was performed in the statistical analysis plan. With nearly equal numbers of patients with normal LV function and those with LV dysfunction in our sample, our study was designed to have 80% 10, with alpha of 0.01 (to account for the multiple hypotheses), to detect a small effect size of 0.25 or more.

## Results

A total of 813 patients were included in the study. Of these patients, 227 (28%) had their echo within seven days of the index surgery, 360 (44%) had their echo within 30 days of the index surgery and only 108 (13%) had their echo 365 or more days before the index surgery.

**Fig 2:**
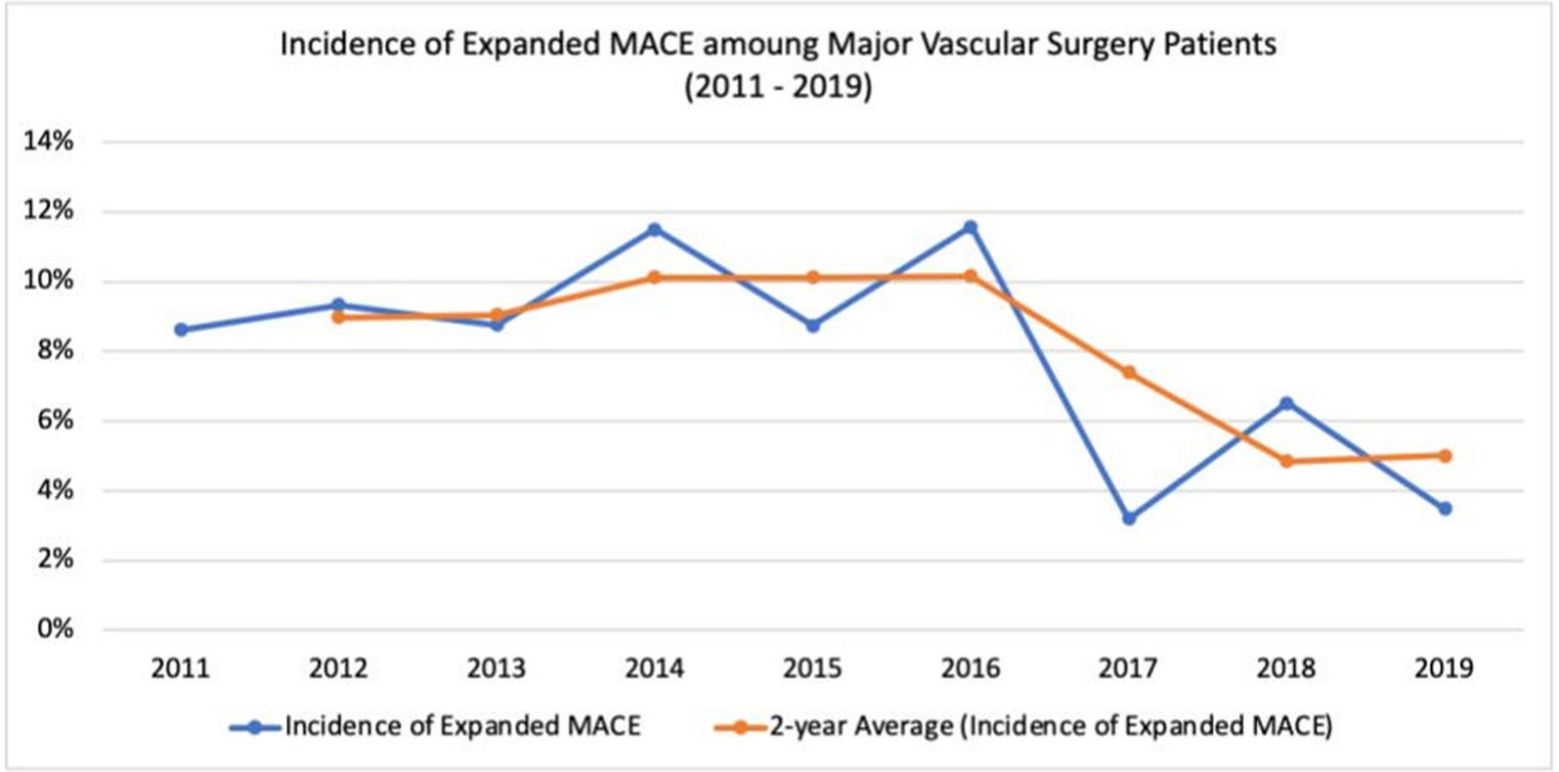
Incidence of Expanded MACE in major vascular surgery patients. At the start of the study period (2011) the incidence of Expanded MACE was 9% and declined to 5% for the last full year of data (2019). MACE: Major adverse cardiovascular events.

Majorities of patients were male (n = 553; 68%), received an ASA score of 3 (n = 524, 64.9%), and had a history of smoking (n = 698, 86.2%) (Table 1). The median age was 67 (IQR: 60–75). CEA was the most common surgery type (n = 243; 29.9%), followed by infrainguinal arterial reconstruction (n = 192; 23.6%), EVAR (n = 124; 15.3%), Complex EVAR/TEVAR (n = 115; 14.1%), suprainguinal arterial reconstruction (n = 98; 12.1%), and open AAA (n = 41; 5.0%). Emergent procedures were 4.3% of all procedures. Procedures were relatively evenly distributed between the years (min: 2011, n = 58, 7.1%; max: 2005 n = 103, 12.7%). The incidence of Expanded MACE decreased from 2011 to 2020 (see Fig 2).

**Table 1:** Clinical characteristics of the sample. AAA: Abdominal aortic aneurysm. AR: Arterial reconstruction. CABG: Coronary artery bypass graft. CEA: Carotid endarterectomy. COPD: Chronic obstructive pulmonary disease. ESRD: End-stage renal disease. EVAR: Endovascular aneurysm repair. TEVAR: Thoracic endovascular aortic repair.

Variable	Level	N (%)	Mean (STD)
DEMOGRAPHICS			
**Age (years)**		813	67 (60, 75)
**Sex**	**Male**	553 (68.0%)	N/A
**Race**	**Asian**	4 (0.5%)	N/A
	**Black**	107 (13.2%)	N/A
	**Caucasian**	689 (85.1%)	N/A
	**Unknown**	10 (1.2%)	N/A
**Hispanic/Latino**	**Yes**	3 (0.5%)	N/A
**ASA Classification**	**2**	41 (5.1%)	N/A
	**3**	524 (64.9%)	N/A
	**4**	241 (29.9%)	N/A
	**5**	1 (0.1%)	N/A
MEDICAL HISTORY			
**Myocardial Infarction**	**Yes**	172 (21.3%)	N/A
**Stable Angina**	**Yes**	117 (14.5%)	N/A
**CABG and/or Cardiac Stent**	**Yes**	305 (37.8%)	N/A
**Congestive Heart Failure**	**NYHA 1**	70 (8.7%)	N/A
	**NYHA 2**	45 (5.6%)	N/A
	**NYHA 3**	20 (2.5%)	N/A
	**NYHA 4**	8 (1.0%)	N/A
**Diabetes Mellitus**	**Yes**	245 (30.5%)	N/A
**COPD**	**Yes**	203 (25.2%)	N/A
**Smoking**	**Never**	112 (13.8%)	N/A
	**History / Current**	698 (86.2%)	N/A
**Cerebrovascular Accident**	**Yes**	140 (35.2%)	N/A
**Carotid Stent or CEA**	**Yes**	59 (7.3%)	N/A
**Prior Aneurysm Repair**	**Yes**	73 (9.0%)	N/A
**Hypertension**	**Yes**	709 (88.3%)	N/A
**ESRD on Dialysis**	**Yes**	23 (2.8%)	N/A
**Creatinine (mg/dL)**		786	1 (0.8, 1.2)
SURGICAL VARIABLES			
**Type of Surgery**	**CEA**	243 (29.9%)	N/A
	**Complex EVAR**	115 (14.1%)	N/A
	**TEVAR**	124 (15.3%)	N/A
	**Infrainguinal AR**	192 (23.6%)	N/A
	**Open AAA**	41 (5.0%)	N/A
	**Suprainguinal AR**	98 (12.1%)	N/A
**Year of Procedure**	**2011**	58 (7.1%)	N/A
	**2012**	75 (9.2%)	N/A
	**2013**	80 (9.8%)	N/A
	**2014**	87 (10.7%)	N/A
	**2015**	103 (12.7%)	N/A
	**2016**	95 (11.7%)	N/A
	**2017**	94 (11.6%)	N/A
	**2018**	92 (11.3%)	N/A
	**2019**	86 (10.6%)	N/A
	**2020**	43 (5.3%)	N/A
**Emergency Surgery**	**Yes**	35 (4.3%)	N/A
**Procedure Time (min)**		753	201 (135, 299)
**Estimated Blood Loss (mL)**		714	150 (75, 300)
**Intraoperative Fluid Resuscitation (mL)**		673	1800 (1257, 3000)
**Intraoperative Vasopressor Infusions**	**≤ 1**	739 (91.5%)	N/A
	**≥ 2**	69 (8.5%)	N/A
**Post-Surgical Length of Stay (days)**		803	4 (IQR: 2, 7)

### Left ventricular function

Of the 812 patients in the sample with an echocardiographic study assessing LV function (Table 2): 400 (49.3%) had LV dysfunction. There was no significant difference in Expanded MACE between the patients who had LV dysfunction (8.3%) and those that did not (7.0%; p = 0.496) (Table 3). Nor was there any significant difference between any of the individual components of Expanded MACE between the two groups. LV dysfunction was not significantly associated with any of the secondary outcomes, although we observed an increased relative risk of postoperative dialysis which did not reach significance (IRR: 6.7, 95%CI 0.8–54.6, p = 0.075).

**Table 2:** Echocardiographic parameters defining left ventricular dysfunction, right ventricular dysfunction, and valvular lesions.

Left Ventricle		Right Ventricle		Valves	
Assessment	Criteria for Dysfunction	Assessment	Criteria for Dysfunction	Assessed	Criteria for Lesion
Qualitative	Mild Moderate Severe	Qualitative	Mild Moderate Severe	Aortic Stenosis	Moderate Severe
Ejection Fraction	<50%			Aortic Regurgitation	Moderate Severe
Diastolic Function	Grade 1 Grade 2 Grade 3			Mitral Stenosis	Moderate Severe
				Mitral Regurgitation	Moderate Severe
				Tricuspid Regurgitation	Moderate Severe

**Table 3:** Primary and secondary outcomes of major vascular surgery patients by echocardiographic findings. Unadjusted logistic (OR) or Poisson (IRR) regression was performed. RV dysfunction was significantly associated with the incidence of postoperative dialysis. IRR: Incidence rate ratio. LV: Left ventricular. N/A: Not applicable. OR: Odds ratio. RV: Right ventricular. *Demonstrates significance of p < 0.05. **Discharge disposition is considered abnormal if it is to any place other than home.

Outcome	Cohort (n = 813)	LV Function	Abnormal (n = 412)	Normal (n = 400)	RV Function	Abnormal (n = 85)	Normal (n = 691)	Valvular Lesion	Present (n = 69)	Absent (n = 737)
**Expanded Major Adverse Cardiac Event**	**62 (7.6%)**	**Y**	34	28	**Y**	5	53	**Y**	2	60
		**N**	377	372	**N**	80	637	**N**	67	676
		**OR [CI]**	1.198 [0.712–2.015]		**OR [CI]**	0.751 [0.434–9.62]		**OR [CI]**	0.336 [0.080–1.407]	
		**P value**	0.496		**P value**	0.553		**P value**	0.136	
**Cerebrovascular Accident**	**11 (1.4%)**	**Y**	4	7	**Y**	2	8	**Y**	1	10
		**N**	392	385	**N**	80	662	**N**	64	707
		**IRR [CI]**	0.57 [0.71–2.02]		**IRR [CI]**	2.04 [0.43–9.62]		**IRR [CI]**	1.11 [0.14–8.62]	
		**P value**	0.496		**P value**	0.366		**P value**	0.925	
**Myocardial Infarction**	**14 (1.7%)**	**Y**	9	5	**Y**	1	12	**Y**	0	14
		**N**	388	387	**N**	81	659	**N**	65	704
		**IRR [CI]**	1.78 [0.60–5.30]		**IRR [CI]**	0.68 [0.09–5.24]		**IRR [CI]**	0 [0.00–0.00]	
		**P value**	0.303		**P value**	0.713		**P value**	1.00	
**Dysrhythmia**	**37 (4.6%)**	**Y**	22	15	**Y**	2	32	**Y**	1	37
		**N**	376	377	**N**	80	640	**N**	64	683
		**OR [CI]**	1.47 [0.75–2.88]		**OR [CI]**	0.50 [0.12–2.13]		**OR [CI]**	0.30 [0.04–2.20]	
		**P value**	0.26		**P value**	0.348		**P value**	0.234	
**Congestive Heart Failure**	**1 (0.1%)**	**Y**	0	1	**Y**	0	1	**Y**	0	1
		**N**	397	390	**N**	82	669	**N**	65	716
		**IRR [CI]**	0 [0.00–0.00]		**IRR [CI]**	0 [0.00–0.00]		**IRR [CI]**	0 [0.00–0.00]	
		**P value**	0.997		**P value**	0.999		**P value**	1.00	
**Cardiovascular Death**	**10 (1.2%)**	**Y**	6	4	**Y**	3	7	**Y**	1	9
		**N**	406	396	**N**	82	684	**N**	68	728
		**IRR [CI]**	1.46 [0.41–5.16]		**IRR [CI]**	3.48 [0.90–13.5]		**IRR [CI]**	1.19 [0.15–9.37]	
		**P value**	0.56		**P value**	0.07		**P value**	0.871	
**Dialysis**	**8 (1.0%)**	**Y**	7	1	**Y**	3	5	**Y**	0	8
		**N**	261	256	**N**	54	438	**N**	44	471
		**IRR [CI]**	6.71 [0.83–54.56]		**IRR [CI]**	4.66 [1.11–19.5]		**IRR [CI]**	0 [0.00–0.00]	
		**P value**	0.075		**P value**	0.035*		**P value**	1.00	
**Respiratory Complications**	**27 (3.3%)**	**Y**	14	13	**Y**	3	23	**Y**	2	25
		**N**	268	253	**N**	56	439	**N**	45	473
		**OR [CI]**	1.01 [0.46–2.20]		**OR [CI]**	1.02 [0.30–3.52]		**OR [CI]**	0.84 [0.19–3.67]	
		**P value**	0.967		**P value**	0.972		**P value**	0.818	
**Hospital Mortality**	**12 (1.5%)**	**Y**	6	6	**Y**	3	8	**Y**	1	11
		**N**	406	394	**N**	82	683	**N**	68	726
		**IRR [CI]**	0.97 [0.31–3.01]		**IRR [CI]**	3.05 [0.81–11.5]		**IRR [CI]**	0.97 [0.13–7.52]	
		**P value**	0.959		**P value**	0.1		**P value**	0.978	
Discharge Disposition**	**180** **(22.1%)**	**Y**	91	89	**Y**	18	153	**Y**	10	168
		**N**	320	311	**N**	66	538	**N**	58	569
		**OR [CI]**	N/A		**OR [CI]**	N/A		**OR [CI]**	N/A	
		**P value**	N/A		**P value**	N/A		**P value**	N/A	

Post-hoc analysis evaluating LV diastolic dysfunction found no difference in primary or secondary outcomes between patients with diastolic dysfunction (n = 302) and those without (n = 160).

### Right ventricular function

Of the 776 patients in the sample with an echocardiographic study assessing right ventricular function (Table 2): 85 patients (11.0%) had RV dysfunction. There was no significant difference in Expanded MACE between the patients who had RV dysfunction (5.9%) and those that did not (7.7%; p = 0.553) (Table 3). Nor was there any significant difference between any of the individual components of Expanded MACE between the two groups. RV dysfunction was associated with an increased risk of postoperative dialysis (IRR: 4.7, 95% CI 1.1–19.5, p = 0.035). RV dysfunction was not significantly associated with any of the other secondary outcomes, although we observed an increased relative risk of postoperative cardiovascular death which did not reach significance (IRR: 3.5, 95%CI 0.9–13.5, p = 0.07).

Post-hoc analysis evaluating patients with RV dysfunction found no difference in primary or secondary outcomes between patients with mild RV dysfunction (n = 58) compared to those with moderate and severe RV dysfunction (n = 27).

### Valvular lesions

Of the 806 patients in the sample with an echocardiographic study assessing valvular function (Table 2): 69 patients (8.6%) had valvular pathology. There was no significant difference in Expanded MACE between the patients who had valvular pathology (2.9%) and those that did not (8.2%; p = 0.136) (Table 3). Neither was there any significant difference between any of the individual components of Expanded MACE nor other secondary outcomes between the patients who had valvular pathology and those that did not.

## Discussion

We analyzed outcomes after vascular surgery over a ten-year period in an academic, tertiary referral center in the United States for patients who had an echocardiographic assessment within two years of their index surgery. We compared the outcomes of patients with ventricular and valvular pathology to those without. We found no significant association between ventricular dysfunction or valvular pathology and the primary outcome of Expanded MACE.

The lack of association between major echocardiographic findings and postoperative MACE may relate to: 1) the transition from open vascular surgeries to endovascular surgeries, 2) improved and rigorous, evidence and guideline-based preoperative cardiac screening, and 3) improvements in the preoperative optimization of vascular surgery patients. The continuation of these improvements in the perioperative care of vascular surgery patients may be why we identified a trend towards a lower incidence of MACE at the end of our study period than the beginning.

### Left ventricular function

Our study results contradict the historical association of LV dysfunction with worse vascular surgery outcomes [16–18]. In 1988, long-term mortality (410 days +/- 390) from major vascular surgery was 40%; for patients with an LV ejection fraction less than 30% long-term mortality was 59% [18]. In 1995, one of the first studies using echocardiography to predict cardiovascular outcomes in vascular surgery patients found an LV ejection fraction less than 50% had a sensitivity of 78% for predicting cardiac complications, and one in four patients with an LV ejection fraction less than 50% had cardiovascular complications [16]. Published in 2010, another study used echocardiography to evaluate cardiovascular outcomes in vascular surgery patients and found LV dysfunction increased cardiovascular events in open vascular surgeries [17]. Similarly, in 2009, LV diastolic dysfunction assessed by transesophageal echocardiography was associated with increased postoperative congestive heart failure following vascular surgery [19]. A post-hoc analysis of diastolic dysfunction found no association with Expanded MACE in our sample.

The absence of a significant association between LV dysfunction and MACE in our study may relate, in part, to the evolution of vascular surgery to a class of less morbid procedures utilizing endovascular techniques. From 2002 to 2008 Flu et al. enrolled patients undergoing vascular surgery and reported that 50% of patients in their cohort had LV dysfunction [17]. The 30-day mortality in this report (2.9%) was nearly twice that in our cohort (in-hospital mortality of 1.5%); perhaps related to a higher proportion of open AAA (25% to 5%). Asymptomatic LV dysfunction was only associated with worse cardiac outcomes for patients who had open vascular surgery, not for endovascular cases [17].

Vascular surgery has changed with the adoption of endovascular approaches previously performed more invasively. Between 2003 and 2013 the number of open AAA performed on Medicare patients declined by 76% while the number of EVARs (including branched and fenestrated) increased by 67% [20]. In the same analysis, open AAA procedures and EVARs exhibited an in-hospital mortality rate of >10% and 2–3%, respectively. Likewise, during the study period (2011–2020), our institution saw a relative decrease in open AAA compared to EVAR of 25%.

Preoperative care and anesthesia techniques have similarly evolved over this period with a focus on preoperative management of cardiac pathology. The American College of Cardiology published, most recently in 2014 [13], evidence-based guidelines for preoperative cardiovascular screening and recommendations for optimization. In the endovascular sub-group of a study which recruited patients from 2002–2008, echocardiographic LV dysfunction was associated with worse cardiovascular outcomes only in symptomatic patients [17]. Current preoperative practice would seek to optimize any symptomatic patient prior to elective, vascular surgery [21].

Additionally, we now have decades of cardiovascular outcome data from vascular surgery and these have been synthesized into recommendations and best-practices for anesthesiologists [21]. Over the past decade the percentage of anesthesia-related adverse events in all surgeries identified in the National Anesthesia Clinical Outcomes Registry decreased to just 4.8% [22].

### Right ventricular function

We identified no significant difference between RV function and RV dysfunction in our primary outcome of Expanded MACE. Our findings are in contrast to a small (n = 108), single-center study analyzing RV dysfunction and outcomes in vascular surgery. This study found an association between RV dysfunction and MACE but no association with cardiovascular mortality [23]. Conclusions were limited by the very small subcohort of patients with RV dysfunction (n = 10).

The only statistically significant finding in our study was an association between RV dysfunction and an increased risk of postoperative dialysis. Due to the small number of dialysis outcomes, we were unable to adjust to any other differences in RV function groups. Still, this finding is pathophysiologically plausible as RV failure can cause a reduction in cardiac output resulting in decreased renal blood flow [24] and increased venous congestion [25] but is inconsistent with a prior small cohort study demonstrating an association between RV dysfunction and mortality, but not with dialysis [26]. However, even though RV dysfunction has been prospectively predictive of mortality in patients with chronic kidney disease [27], research into the relationship between pulmonary hypertension, RV dysfunction and chronic kidney disease is in its infancy [28].

### Valvular pathology

We identified no significant difference in the incidence of Expanded MACE between patients with normal or mild compared to those with moderate or severe valvular dysfunction. This finding is consistent with that of *Ouriel et al*. who found valvular abnormalities on echocardiography to be a “poor predictor of cardiac complications” in vascular surgery patients [16] but at odds with a larger multicenter retrospective study (n = 26,231) that associated valvular disease with 30-day postoperative myocardial infarction in vascular surgery [3]. Neither study identified the affected valves, defined the type of valvular dysfunction (stenosis v. regurgitation), or the grade of valvular lesions.

### Clinical implications

Vascular surgery patients should still receive echocardiography as deemed necessary by presentation. Echocardiographic findings in vascular surgery patients should be contextualized by severity of the surgical problem and the patient’s medical history. It is likely knowledge of cardiac limitations provided by echocardiography allow anesthesiologists and surgeons to adjust their treatments and minimize potential harm; well-designed clinical trials are needed to elucidate this nuance. Still, perioperative clinicians should be aware that echocardiographic findings classically considered clinically concerning for vascular surgery are not necessarily associated with worse clinical outcomes.

The true clinical implications of this study will be borne out by future research. Our results require confirmation in larger, multicenter studies and must assess meaningful long-term outcomes such as quality of life, functional status, MACE and mortality. Efforts could be directed towards understanding whether echocardiographic evidence of cardiac dysfunction is associated with MACE in patients undergoing open vascular surgical procedures. Of particular interest is the role of the RV in cardiac outcomes after vascular surgery, given our hypothesis-generating findings.

### Limitations

Selection bias is likely. Study design required echocardiographic data within two years of the index procedure, an inclusion criterion which may skew the cohort towards more severe cardiac disease. It is also possible some patients were not offered vascular surgery due to the severity of their cardiac pathology. Investigators only had access to echocardiogram reports available through the UVA Health electronic medical record. If a patient had an echocardiographic study performed elsewhere and not submitted to the UVA electronic medical record, the case would have been excluded.

While it is the practice at our institution to document an echocardiogram following cardiac surgery, it is possible patients in this sample could have had an echo at our institution, had their cardiac surgery (eg. valvuloplasty) at another institution, and then had their vascular surgery at our institution and been included in this study. We did not capture the clinical symptoms prompting the patient’s echocardiography. We were also limited by our database which did not have details on pre-admission hyperlipidemia or atrial fibrillation and did not have long-term outcomes. Finally, although the study cohort was relatively large, it was limited to a single tertiary care hospital and the possibility exists that practice and related outcomes could differ substantially at other centers.

## Conclusion

We identified no significant difference in Expanded MACE after vascular surgery between patients with or without LV dysfunction, RV dysfunction, or moderate to severe valvular dysfunction. We identified a significant association between RV dysfunction and post-operative dialysis that should be interpreted carefully due to the small number of outcomes and inability to risk-adjust. We conclude that the transition from open to endovascular surgery and advances in perioperative preparation and management have led to improved cardiovascular outcomes. Echocardiographic findings such as LV dysfunction, RV dysfunction and moderate to severe valvular lesions may not be clinically predictive of cardiovascular outcomes given the advances in preoperative preparation, as well as anesthetic and surgical techniques.

## Supporting information

S1 File (PDF)
