# Supplementary material for: Clinical implications of preoperative echocardiographic findings on cardiovascular outcomes following vascular surgery: An observational trial
Source: PLoS One. 2023 Jan 19;18(1):e0280531. doi: 10.1371/journal.pone.0280531 (PMC9851553; doi:10.1371/journal.pone.0280531)
Supplement: S1 File — (PDF) [file pone.0280531.s001.pdf]

Echocardiography as a Risk-Assessment for Major Vascular Surgery Outcomes

Statistical Analysis Plan
(SAP)

IRB: 20906

SAP Authors: Matthew J. Meyer, MD
Sarah J. Ratcliffe, PhD

SAP Version: 1.0

SAP Date: March 25, 2021

1. Description

Patient with coronary artery disease (CAD), heart failure and abnormal heart function undergoing major vascular surgery have a high associated high morbidity and mortality with myocardial infarction accounting for 33-50% of perioperative deaths. [1-4] The prevalence of CAD in vascular surgery patients is high. Proper pre-procedure protocols to accurately assess patients and determine who may require further medical optimization prior to undergoing surgery help mitigate risk and improve outcomes. We designed this study as a single center retrospective cohort analysis to explore the association between ventricular (LV and RV function) and valvular (Aortic / Mitral / Tricuspid) function and expanded major adverse cardiac events (X-MACE).

2. Hypotheses

H1: Depressed left ventricular function is associated with worse clinical outcomes in major vascular cases

H2: Depressed right ventricular function is associated with worse clinical outcomes in major vascular cases

H3: Moderate or severe valvular lesions are associated with worse clinical outcomes in major vascular cases

3. Design

3.1. Study Type

This is an observational study of major vascular patients.

3.2. Blinding

No blinding is involved in this study

3.3. Study Design

This is a retrospective cohort study. Eligible subjects are those who had major vascular surgery at UVA Health and an echocardiography within two years of the index surgery. Major vascular surgery was defined as: supra-inguinal and infra-inguinal vascular bypass and endarterectomy, carotid endarterectomy, open aortic repair, endovascular aneurysm repair (EVAR), thoracic endovascular aortic repair (TEVAR), other complex aortic surgeries (e.g. FEVAR).

If a subject had any additional qualifying vascular procedure within 30 days of the index operation, this was NOT included.

4. Sampling Plan

4.1. Existing Data

SAP registration will occur prior to analysis of the data.

4.2. Data Collection Procedures

Data for this study came from three databases: 1) UVA Health Vascular Quality Initiative (VQI) database, 2) UVA Department of Anesthesia Data Perioperative Informatics database, and 3) echocardiography database extracted from EPIC electronic medical records.

Patient selection began with all subjects in the UVA Health VQI database who had “major vascular surgery” with a date of surgery of January 2011 or later (end date of June 2020). All vascular surgery patients are captured in the UVA Health VQI database.

Inclusion criteria included: echocardiography from less than two years prior to index surgery. Exclusion criteria included: additional vascular surgery occurring within 30 days of a vascular surgery that was included in the database. Intraoperative data was extracted and added to the database from the UVA Department of Anesthesia Perioperative Informatics.

5. Sample Size

The sample size was determined by all eligible patients captured in the UVA VQI database (starting January 2011) who had an echocardiography performed within two years of index surgery.

CEA first case: June 2011
EVAR first case: Jan 2011
Infra first case: May 2011
oAAA first case: April 2011
Supra first case: May 2011
Complex first case: Jan 2012

For a binary primary outcome with a binary exposure, Table 5.1 shows the sample size needed to find statistical differences under a range of effect sizes and exposure group allocations, with an assumed alpha level of 0.01 and 80% power.

Table 5.1: Sample size required for a two-sided test of proportions with alpha=0.01 and 80% power

Percentage in Exposure Group 1	Percentage in Exposure Group 2	Effect Size	Sample Size
10	90	0.2 (Small)	3,250
		0.5 (Medium)	520
		0.8 (Large)	210
25	75	0.2 (Small)	1,560
		0.5 (Medium)	252
		0.8 (Large)	100
50	50	0.2 (Small)	1,168
		0.5 (Medium)	188
		0.8 (Large)	74

6. Variables

6.1. Outcome Variables

The primary outcome variable is:

- Expanded MACE. This is a composite outcome defined as any cardiovascular death, non-fatal MI, non-fatal stroke, post-operative CHF, or new dysrhythmias, all within the index hospital admission.

Secondary outcomes are:

- Cardiovascular death within index hospital admission.
- Non-fatal MI within index hospital admission.
- Non-fatal stroke within index hospital admission.
- Post-operative CHF within index hospital admission.
- New dysrhythmias within index hospital admission.
- Hospital length of stay (LOS); defined as date of surgery to discharge
- In-Hospital mortality

Exploratory outcomes are:

- Discharge location (Home v. Not Home)
- Respiratory complications
- Post-op Temporary / Permanent Dialysis (not counted if on dialysis pre-op)

6.2. Exposure Variables

The exposure variables of interest are:

H1: Depressed left ventricular function is associated with worse clinical outcomes in major vascular cases

- LV ejection fraction < 50%
- LV systolic function; defined as mild / mod / sev decreased
- LV diastolic function; defined as mild (g1) / mod (g2) / sev (g3) decreased

H2: Depressed right ventricular function is associated with worse clinical outcomes in major vascular cases

- RV systolic function; defined as mild / mod / sev decreased

H3: Moderate or severe valvular lesions are associated with worse clinical outcomes in major vascular cases

- AS / AR / MS / MR / TR with moderate or severe dysfxn

6.3. Other Variables

Other variables to be included in the analyses are:

Continuous variables:

- Age
- Creatinine (pre-op)
- Total procedure time

- EBL
- Total fluids transfused (crystalloids, colloids)
- FiO2 mean level
- Year of Surgery
- Weight

Categorical variables:

- Type of Surgery
- Gender (1=male)
- Race (2=Asian, 3=black, 5=white, 7=unknown)
- Hispanic or Latino (1=yes)
- Smoking (0=never, 1=prior/current)
- Hypertension (0=no, 1=yes)
- Diabetes (0=no meds, 1=meds)
- Prior CABG/PCI (0=no, 1=yes)
- ASA (scale)
- Emergency (0=no, 1=urgent/emergent)
- Prior CHF (0=no/asymptomatic, 1=some limitation of physical activity)
- Prior CVA/CVD/Neuro symptoms (0=no, 1=yes)
- Prior CEA/CAS (0=no, 1=yes)
- Dialysis (0=no, 1=yes)
- Intra-operative vasopressor (0 or 1 drip v. 2+ drips)

7. Analysis Plan

7.1. Examination of Baseline Characteristics

Standard descriptive statistics will be used to describe baseline characteristics at the time of surgery, both overall and within each exposure group. Summary statistics such as medians, ranges, minima and maxima, and percentages will be produced for all measured variables. Frequencies will be computed for all categorical and ordinal variables. Graphical methods including histograms, scatterplots, boxplots, and lowess plots, will be used in order to understand aspects of data quality, examine assumptions (such as normality) underlying statistical models, identify potential influential points, and guide in the choice of transformations if warranted.

7.2. Analysis of Primary Outcome

The primary outcome will first be compared within each exposure group using logistic regression. Multivariable logistic regression will be used to test for associations after adjusting for other variables. Penalized logistic regression (lasso) will be used to determine the important variables to be included in the adjusted models.

Before any regression models are constructed, all variables to be adjusted for will be checked for collinearity with each other and the exposure variables using the condition index and Spearman correlation matrix. Any variable deemed to be strong correlated (rho>0.7) with an

exposure variable will be carefully evaluated and considered for removal from the potential set of adjustment variables. All other variables will be allowed to enter the models.

Since there are three primary hypotheses of interest, an alpha level of 0.01 will be used for statistical significance for the associations between the exposures and the outcome.

7.3. Analysis of Secondary Outcomes
Secondary outcomes will be compared within each exposure group following similar methods as the primary outcome. Binary outcomes will use logistic regression, time-to-event outcomes (LOS) will use Cox regression.

7.4. Analysis of Exploratory Outcomes
Outcomes will be compared within each exposure group following similar methods as the primary outcome. Binary outcomes will use logistic regression, continuous outcomes will use linear regression.

7.5. Missing Data
Observational data is likely to have occasional missing data. The possibility of missing data can have serious consequences for the interpretation of statistical analyses. This is particularly problematic if the probability of missing data is related to the exposure, the outcome, or both. We will characterize the amount (%) and pattern of missing data for all variables, and compare the distribution of other variables by those with and without missing data. Should the amount of missing data be >5% or difference found between those with and without missing data, multiple imputation will be used to estimate the missing values.
